# From Something Old to Something New: Functionalist Lessons for the Cognitive Science of Scientific Creativity

**DOI:** 10.3389/fpsyg.2021.750086

**Published:** 2022-01-17

**Authors:** Guilherme Sanches de Oliveira

**Affiliations:** Biological Psychology and Neuroergonomics, Technische Universität Berlin, Berlin, Germany

**Keywords:** creativity, representation, mind, experience, functionalism, embodied cognitive science

## Abstract

An intuitive view is that creativity involves bringing together what is already known and familiar in a way that produces something new. In cognitive science, this intuition is typically formalized in terms of computational processes that combine or associate internally represented information. From this computationalist perspective, it is hard to imagine how non-representational approaches in embodied cognitive science could shed light on creativity, especially when it comes to abstract conceptual reasoning of the kind scientists so often engage in. The present article offers an entry point to addressing this challenge. The scientific project of embodied cognitive science is a continuation of work in the functionalist tradition in psychology developed over a century ago by William James and John Dewey, among others. The focus here is on how functionalist views on the nature of *mind, thought*, and *experience* offer an alternative starting point for cognitive science in general, and for the cognitive science of scientific creativity in particular. The result may seem paradoxical. On the one hand, the article claims that the functionalist conceptual framework motivates rejecting mainstream cognitive views of creativity as the combination or association of ideas. On the other hand, however, the strategy adopted here—namely, revisiting ideas from functionalist psychology to inform current scientific theorizing—can itself be described as a process of arriving at new, creative ideas from combinations of old ones. As is shown here, a proper understanding of cognition in light of the functionalist tradition resolves the seeming tension between these two claims.

## 1. Introduction

Innovative design often involves combining previously separate features or functions into a single new product. Obvious examples include simple 2-in-1 products, such as the shampoo/conditioner combo, the washing machine with a built-in dryer, and the versatile baby car seat that transforms into a stroller. This is also the case with multi-functional (i.e., many-in-one) products, such as current cellphones: in addition to making phone calls, the smartphone also acts as a camera, GPS device, calculator, alarm clock, internet browser, and more. What was creative about products like these when they were first invented was not that they did something entirely novel that did not exist previously: rather, their originality lies in bringing together in a single object different uses that were already well known but which had previously been separate from one another.

From a psychological standpoint, creativity is frequently described in a similar fashion as relating to the production of novelty out of what was already familiar. Some researchers have emphasized the role of personality traits in lowering barriers to creative behaviors: different levels of openness, extraversion, nonconformity, impulsivity, and persistence, for instance, might increase the likelihood of diverging from more familiar behaviors or increase the occurrence of unusual associations (refer to, e.g., Batey and Furnham, 2006; Feist, 2010, 2019 for detailed reviews). Other researchers have emphasized social psychological factors contributing to creative performance, including evaluation by others and expectations of future evaluation, the availability of rewards, and access to role models to draw from in generating original behavioral variations (refer to, e.g., Hennessey, 2003, 2010; Amabile et al., 2018). From a specifically cognitive focus, research on imagination has shown that when we try to come up with a completely novel member of a given category, properties that are typical for members of that category tend to structure our imagination (Ward, 1994; Ward et al., 1999; Ward and Kolomyts, 2010). Even when we cannot trace the origin of a new thought, idea, or behavior, the process underlying creative insight is usually assumed to involve some kind of combining or associating prior knowledge in a new way rather than actually coming up with something completely novel out of thin air. As Thomas Ward puts it, “Clearly, ideas do not arise in a vacuum, and there is a trivial sense in which we must always rely on some type of stored information when we develop any new idea” (Ward, 1995, p. 157–158).

The present article is concerned with evaluating this intuitive view of creativity in terms of combinations or associations, even in this supposedly trivial sense. The special focus is on how the combinatorial view figures in the *cognitive science of science*, that is, in the application of theories and methods from cognitive science toward investigating cognitive phenomena at play in scientific practice itself. The dominant paradigm in cognitive science is to understand the mind in terms of the internal knowledge structures and procedures that mediate between perceptual inputs and behavioral outputs (refer to, e.g., Neisser, 1967/2014; Marr, 1982; Fodor, 1983; Pinker, 1997; Thagard, 2005, 2019; Boden, 2008; Clark, 2014). Along these lines, cognitive explanations of scientific practice have tended to emphasize the role of mental representations and computational information-manipulating procedures in enabling the individual scientist to generate new knowledge (refer to, e.g., Gholson et al., 1989; Gopnik, 1996; Carruthers et al., 2002; Feist, 2008; Nersessian, 2008; Feist and Gorman, 2012; Thagard, 2012).

In parallel with the dominant computational-representational approach, an increasingly popular alternative understands cognition as an embodied, embedded, extended, enactive, and ecological phenomenon (refer to, e.g., Gibson, 1979; Maturana and Varela, 1980; Varela et al., 1991; Clark, 1998, 2003, 2008; Calvo and Gomila, 2008; Chemero, 2009; Menary, 2010; Shapiro, 2010, 2014; Di Paolo et al., 2017; Gallagher, 2017; Newen et al., 2018). This diverse research tradition, referred to as *embodied cognitive science* or “4E cognition” for short, is widely recognized (even by critics) as being very successful in explaining non-representationally so-called “lower-level” behaviors, which includes, for instance, accounts of how we navigate cluttered environments without collision or how we catch balls and other moving targets, all in terms of real-time perception-action coupling. But, embodied cognitive science appears to face a problem. This is the problem of “scaling up” non-representational, embodied explanations so that they can also make sense of what has been referred to as “representation-hungry” processes, i.e., processes that seem to require explanation in terms of internal representations, such as abstract conceptual thinking, logical reasoning, imagination, memory, and language (Clark and Toribio, 1994; Chemero, 2009; Gallagher, 2017; Sanches de Oliveira et al., 2021).

The scaling-up problem presents a challenge for embodied cognitive science in general, but it can also seem to pose a special barrier for the application of the embodied approach in the cognitive science of science. This is because scientific practice exhibits all of the main characteristics that are usually considered to be problematic for embodied, non-representational approaches to cognition. No doubt, scientists use their bodies to interact with parts of the world that are readily available for perception, as is clear, for instance, in operating equipment to conduct experiments and collect data. But even in these cases, scientific work is unavoidably tangled in a complex web of meaning that seems paradigmatically “representation hungry,” involving reasoning about entities that are not immediately available for real-time perception and that perhaps could not even possibly be available in that way, such as in the case of abstract concepts.

This is the issue that the present paper is ultimately concerned with, namely, examining the view of creativity as a combination or association with an eye to determining how it might fit in the broader project of approaching scientific practice from the perspective of embodied cognitive science. Embodied cognitive science is sometimes treated by critics as a new trend, and the canonical references cited above, mostly from the past couple of decades, illustrate the focus on recent research. In fact, however, embodied cognitive science is rooted in work in the functionalist tradition extending all the way back to the late 19th century and the origins of psychology as a distinct scientific discipline (Heft, 2001; Crippen and Schulkin, 2020). By exploring these functionalist roots of embodied cognitive science, the goal here is to find a new way of understanding creativity that makes it more amenable to explanation in non-representational, embodied terms. Section 2 reviews how, within the computational-representational paradigm, scientific creativity is understood in terms of combination and association. Section 3 examines some of the key elements making up the functionalist approach to psychology as developed by William James and John Dewey. Section 4 then traces out implications of those aspects of the functionalist paradigm for thinking about creativity, especially in science, from the perspective of embodied cognitive science. As its functionalist roots make clear, embodied cognitive science is more than just a local theory limited to “lower-level” cognitive phenomena: rather, it is a comprehensive approach capable of advancing our understanding of all of cognition, including even phenomena of interest for the cognitive science of science.

## 2. Creativity in Computational-Representational Cognitive Science

### 2.1. The Newell, Shaw, and Simon Model and the Foundations of Computational-Representational Cognitive Science

Cognitive approaches to creativity are about as old as cognitive science itself. If the year 1956 is taken as the birth of cognitive science (Miller, 2003), it would only be another couple of years until Newell et al. (1958/1962) offered an early example of a computational approach to creative thinking. They described their approach as follows: “we would have a satisfactory theory of creative thought if we could design and build some mechanisms that could think creatively (exhibit behavior just like that of a human carrying on creative activity), and if we could state the general principles on which the mechanisms were built and operated” (Newell et al., 1958/1962, p. 64). While recognizing that the programs they described fell short from the level of sophistication and complexity characteristic of human creativity, the authors held that the processes at play were of the same type: their working hypothesis was that “creative thinking is simply a special kind of problem-solving behavior” (p. 65) and, however simple, the candidate explanations they offered were “qualitatively of the same genus as these more complex human problem-solving processes” (p. 116). In their view, effective problem solving is a matter of employing “search” strategies that narrow down the entire “problem space” and enable finding acceptable solutions within a restricted time frame. The key to making sense of problem-solving behavior that is effective in a *creative* manner, then, lies in considering the “system of heuristics possessed by the problem-solver” (p. 104). In many cases, when strategies that have been useful in the past prove unsuccessful for dealing with new situations, a problem-solver will be “more likely to succeed if his repertoire of heuristics includes the injunction: ‘If at first you don't succeed, try something counter-intuitive'" (p. 105). Consequently, while in some cases, a creative solution can be found by employing a combination of different heuristics, in other instances it might require “generat[ing] new heuristics to fill the vacuums created by the rejection of the ones previously used” (p. 107).

The focus of this section will be on a different, more recent cognitive account of creativity. Still, starting with Newell, Shaw, and Simon's account is instructive for two reasons.

The first reason has to do with the view of creativity at play in their account. The brief summary given above emphasized their goal of explaining *how* problem-solving behavior can be creative, namely depending on the heuristics that problem solvers know and use. But although their focus was explicitly on the ‘how' of creativity, they did not ignore the “what” question: for them, this was the question of *what* makes a given problem solution *creative* in the first place. Synthesizing the prior psychological literature and bringing it to the nascent domain of cognitive science, Newell, Shaw, and Simon identify four criteria each of which they thought sufficed to make a solution count as creative: “creative activity appears simply to be a special class of problem-solving activity characterized by novelty, unconventionality, persistence, and difficulty in problem formulation” (p. 66). Contemporary work tends to emphasize novelty and unconventionality without seeing persistence and difficulty as nearly as important. Along these lines, for instance, Margaret Boden offers the following characterization: “Creativity is the ability to come up with ideas or artifacts that are new, surprising, and valuable” (Boden, 2004, p. 1). Boden also adds—without reference to Newell, Shaw and Simon, but clearly in agreement with them—that creativity is not a sui generis process, “not a special “faculty” but an aspect of human intelligence in general” (ibid.). Newell, Shaw, and Simon's inclusion of persistence and difficulty is likely motivated by their attention to creative innovation in highly skilled activities in science and technology: as they propose, these require “motivation” to sustain specialized activities “either over a considerable span of time (continuously or intermittently) or at high intensity” (p. 65) and sometimes problems in these specialized domains are “vague and ill-defined, so that part of the task was to formulate the problem itself,” which can be difficult (p. 66). But even Newell, Shaw, and Simon's description of novelty was prescient: in their view, the first criterion for creative problem-solving is that “The product of the thinking has novelty and value (either for the thinker or for his culture)” (p. 65). In this, they seem to anticipate a distinction that would be drawn much later between the “P-creative” and the “H-creative,” that is, between that which is innovative for an individual yet may not be innovative for others and that which is historically innovative, or novel for all (Boden, 1990, 2004; see also, e.g., Nersessian, 2008).

Relatedly, but beyond the specific topic of creativity, a second reason for the relevance of their account for the purposes of this article is that the main theoretical commitments they articulated have remained by and large the same in contemporary cognitive science. What is key here is the assumption that *providing a cognitive explanation of some phenomenon amounts to providing an explanation in computational-representational terms*. Newell, Shaw, and Simon propose: “In order for us to think about something, that something must have an internal representation of some kind, and the thinking organism must have some processes that are capable of manipulating the representation” (Newell et al., 1958/1962, p. 102). The assumption here is that the mind trades in representations of the external world: through perception, we obtain information from the environment, and intelligent behavior arises from manipulating those internal information structures. In some cases, information processing is for planning and executing motor actions that involve continued contact with the environment and typically require further sensory feedback. In other cases, information processing is for engaging in more purely intellectual activities, such as when we think about objects that are no longer present externally and that, presumably, must therefore remain accessible in the form of internally-stored mental representations. In either case, according to this view, a proper understanding of mind and behavior calls for description in terms of computational procedures and the manipulation of internal representations or information structures.

If this way of understanding mind and behavior sounds uncontroversial, that is precisely because the work by people like Newell, Shaw, and Simon helped establish this as the standard way of doing cognitive science. Foreshadowing Marr's (1982) influential distinction between levels of description, in this early paper on creativity Newell et al. (1958/1962, p. 98) already indicate how the project of computational-representational explanation is seen as independent from research on what goes on in the brain: “We are asking here neither a physiological nor a ‘hardware' question. We wish an answer at the level of information processing, rather than at that of neurology or electronics.” Exclusive focus on explanation at the cognitive level without any regard at all to advances in neuroscience is increasingly less common today: for the past couple of decades, cognitive science continues to be “pulled vertically down into the brain,” as Bechtel et al. (1998/2017) put it. But the nature of this shift toward closer contact between cognitive and neural explanations is illuminating. To a large extent, this shift has been possible not so much because, through contact with neuroscientific research, cognitive scientists have begun reconceptualizing cognition in neurophysiological terms—on the contrary, the shift is for the most part due to the increasing popularity of conceptualizations of the brain and neural processes in computational-representational terms (refer to endorsements as well as critical discussions in, e.g., Posner et al., 1988; Sejnowski et al., 1988; Barlow, 1994; Boden, 2008; Neske, 2010; Piccinini and Bahar, 2013; Anderson, 2014; Gazzaniga, 2014; Brette, 2019). The assumption that we find already in Newell, Shaw, and Simon's work and that remains widespread today is that, whatever it does, physiologically speaking, the brain can be adequately understood as engaging in storing and processing information. It was by building upon the foundation of this computational-representational theoretical assumption that the contemporary cognitive neuroscientific project of understanding creativity could become equated to “the question of how brains compute creative ideas” (Dietrich and Haider, 2017, p. 2).

### 2.2. Thagard's Neurocomputational Account of Scientific Creativity

Paul Thagard's approach to creativity, including scientific creativity, exhibits important similarities and dissimilarities to the one by Newell, Shaw, and Simon. Conceptually, Thagard's account agrees in highlighting the role of novelty, surprise, and value in creativity: in his view, something (say, a behavior, a concept or a product) is creative when it is original, when it is unexpected because not obvious, and when it is considered useful and important (Thagard, 2019). Theoretically, Thagard also approaches creativity from a computational-representational perspective and focuses on the “how” question of understanding the information-processing mechanisms involved in the generation of creative outputs. Still, his account differs significantly from the one by Newell, Shaw, and Simon because, in line with the recent developments just described, Thagard's project embraces the more recent idea that the computational-representational framework can be a way of integrating cognitive and neural explanations rather than divorcing the two. Given this, a more fundamental difference lies in the specific mechanism posited to explain creativity. Unlike Newell, Shaw, and Simon's focus on problem solving and on the “creative” as a feature of some problem solutions, Thagard's focus is more general, not limited to problem solving but encompassing other kinds of behaviors and activities understood in reference to neural processes.

This more fundamental difference is made clear by Thagard's claim that he sees “the problem of creative combination of representations as an instance of the ubiquitous ‘binding problem' that pervades cognitive neuroscience” (Thagard, 2012, p. 114). Put briefly, the binding problem is the problem of explaining how we integrate multiple sensory inputs, even from different sensory modalities, into unified representations. In typing these very words, for instance, I feel the keys at the tip of my fingers, I hear the sound of each key press, and I see the letters show up in succession on the screen. And yet, each of these sensory inputs (tactile, auditory and visual) goes on unnoticed—or at least they did until I started writing this example—and the whole situation unfolds smoothly as I have a single coherent experience of the world rather than a jumble of disjointed sensations. This applies even for a single sensory modality. Focusing on vision, Thagard explains: “Recognizing objects requires combinations of features” such that, when a monkey recognizes a banana, that recognition involves binding “[d]ifferent neural groups [that] serve to encode different features of bananas, such as yellow, long, and round” much like the binding of features such as nose, eyes, and mouth when you recognize a friend's face (Thagard, 2019, p. 26). The binding problem, then, is the problem of understanding how this happens. The problem matters for us precisely because, again, in Thagard's account the binding of distinct representations explains not only the unity and coherence of ordinary experience but also the occurrence of behaviors and concepts that are novel, surprising, and valuable, that is, that are creative: as he puts it, “representation combination” or binding “is the fundamental mechanism of creativity in various domains” (Thagard, 2012, p. 142).

Thagard's account is based on what he calls the “combinatorial conjecture,” which he describes as the view that “All creativity results from convolution-based combination of mental representations consisting of patterns of firing in neural populations” (Thagard, 2012, p. 142). The account can thus be understood in light of two distinctions, the first between combinatorial models and non-combinatorial alternatives, and the second between combinatorial models that are based on convolution and combinatorial models that are not.

First, in endorsing the combinatorial conjecture, Thagard (2012) explicitly opposes the alternative view that, rather than involving the combination of different representations, a creative idea is the result of blind variations on a single concept or representation. As he explains, this kind of view is generally inspired by an analogy to evolutionary processes in biology: just as new adaptive biological features arise through random genetic mutations, so are original and useful concepts supposed to arise through mutation or blind variation on prior versions of individual concepts. A more specific target of Thagard's (2012) criticism is the view developed by Simonton (2010) as a reworking of Campbell's (1960) blind-variation and selective-retention (BVSR) model of creativity (refer to, e.g., Simonton, 1999, 2011, 2013, 2018). After examining Simonton's model, Thagard suggests that, even accounting for the role of serendipity and unforeseen developments, scientific discoveries can never be entirely blind. In Thagard's example, although Galileo may not have had the intention to discover the moons of Jupiter, still “his interests, background knowledge, and cognitive processes made it more probable that” upon turning his telescope in the right direction “he would generate the representation ‘Jupiter has moons”' compared to the probability of generating “some utterly useless representation such as ‘Rome has toes' ” (Thagard, 2012, p. 157). This leads Thagard to the conclusion that blind variation cannot be the mechanism for creativity: “discovery is not blind, and biological evolution is a poor model for scientific discovery and other kinds of creativity” (Thagard, 2012, p. 157).

A sticking point for Thagard, therefore, seems to be the notion of “blindness” at play in the BVSR view. Interestingly, however, Simonton explicitly states that blindness does not equate to randomness: “Although all random variations are inexorably blind, blind variations are not inevitably random, and are actually seldom so” (Simonton, 2010, p. 157). Elsewhere Simonton explains that blindness does not consist in the “equiprobability for all possible alternatives” (Simonton, 1999, p. 312); rather, blindness more simply suggests that the variation is not *sighted* in the sense of being informed or guided by the probability of the variation's success. This suggests that Thagard's criticism, as illustrated in his Galileo example, may be unwarranted since Simonton's BVSR model does not entail the equiprobability of the success of both Galileo's discovery and of his hypothetical nonsensical thought. Not only that, but the example also suggests blind variation to be the whole story, when in reality the BVSR model expressly includes a second element of selective retention (this is the “SR” in “BVSR”); and this selective element is something creativity also has in common with biological evolution: “The creative individual, too, produces original ideas, which are then subjected to first cognitive and then sociocultural selection, retaining solely those ideas that are adaptive by some criteria of utility, truth, or beauty” (Simonton, 1999, p. 322; and also, e.g., Simonton, 2010, p. 157). So, although this selective function is not part of the variational process itself (which is blind), it is still present at the (sub)personal and collective levels, which could explain why Galileo did not have something like the nonsensical idea Thagard described, or at least why we would not know about it if he did. Last, besides these points about blindness and the importance of selection, Thagard's focus on Simonton (2010) as a target of criticism is curious for the further reason that, in Simonton's account at least, blind variation is an explicitly *combinatorial* mechanism: rather than the single-concept mutation view, what Simonton proposes is precisely that creativity is “contingent on the capacity to proliferate blind combinatorial variations” (Simonton, 2010, p. 158; refer to, e.g., Simonton, 2011, 2013), which situates both Simonton's and Thagard's accounts within the same larger camp, even if they disagree with regard to the specific nature of combinatorial processes.

Setting aside the question of whether Thagard's (2012) criticism of Simonton's (2010) account is warranted, the second contrast useful for understanding Thagard's proposal is one between his convolution-based combinatorial approach and the alternative combinatorial approach based on neural synchronization. Both approaches, as Thagard explains, are candidate solutions to the binding problem. In the neural synchronization view, the coherence of experience is a result of the binding of neuronal firing patterns that represent different incoming sensory stimuli but that are temporally coordinated: “The neural synchrony hypothesis is that binding takes place because the representations that need to be bound together are firing in the same temporal patterns” (Thagard, 2019, p. 26). Thagard criticizes neural synchronization models for being too biologically implausible, in some cases making assumptions that do not hold about real neurons and in other cases requiring “more neurons than exist in the human brain” (Thagard, 2012, p. 115). He also identifies important cognitive limitations attending synchronization models (refer to Thagard, 2019, pp. 26–27). On the one hand, for instance, while temporal coordination may contribute to how we recognize perceived objects through binding sensory inputs, Thagard suggests it is not clear how this can accommodate the fact that we often draw inferences about those perceived objects for which there is no incoming sensory stimulation. On the other hand, even if neural synchronization is responsible for the binding of representations that are temporally coordinated, Thagard argues that neural synchronization models do not thereby explain how we manipulate representations once they have been bound together. Limitations like these pose general challenges for neural synchronization as a solution to the binding problem, but they are even more relevant for the project of understanding the emergence of creative thoughts and ideas, since in the combinatorial perspective these are supposed to arise from manipulations that bind together different representations. These limitations motivate Thagard to pursue an alternative approach drawing instead on Eliasmith's (2013) work on convolution and semantic pointers.

Thagard offers two helpful analogies to explain the perspective he favors. The first analogy describes convolution by reference to braiding hair. If you weave together strands of hair to form a braid, later it is still possible to undo the braid to get the strands back apart; similarly, convolution is a mathematical function by which neuronal patterns are hypothesized to weave together representations in such a way that it is still possible to access the individual representations separately (which would not be the case if concepts always mutated like genes). The second analogy describes the semantic pointer architecture by analogy to street addresses. An address with street name and building number offers a short and convenient way to represent whatever occupies a specific location (e.g., your house); similarly, a pointer in a computational sense is a representation whose value is the value of some other representation or set of representations, such that it functions as an address that indicates (i.e., points to) that representation or set. These two notions work together because, in the view Thagard adopts, “Semantic pointers result from binding multiple representations together by means of convolution” (Thagard, 2019, p. 29): that is, through convolution, neural patterns of activity with all kinds of different representational content can be “woven” (or bound) together, regardless of temporal coordination; and by doing so as part of an entire architecture of pointers, the woven or bound pattern can function as a specific pointer without thereby compromising access to the individual representations directly or their use as part of other pointers. It is for reasons like these that Thagard proposes that “creativity results from neural processes that include binding of representations, generation of new concepts and rules, and the application of analogies” (Thagard, 2019, p. 247). As he explains, the binding applies to “the full range of multimodal representations needed for creativity in the domains of scientific discovery, technological invention, artistic imagination, and social innovation” (p. 248): this includes, for instance, verbal, mathematical, imagistic, or sensorimotor representations, all of which can be combined into new pointers that are creative because they are original, surprising, and valuable in the specified domain.

Thagard's account is relevant for us as a prime example of a cutting-edge approach to creativity in the cognitive science of science. Thagard (2012, 2019) is explicitly concerned with the project of applying theories and methods from cognitive science to make sense of science, including scientific practices of problem solving, explanation, modeling, belief formation, conceptual change, and theory development, and it is in the context of this larger project that he offers an account of creativity as it occurs for individual cognitive agents engaged in scientific work. Moreover, Thagard's view is particularly significant for present purposes because of his outspoken criticism of non-representational approaches in embodied cognitive science. Thagard characterizes non-representationalism as “radical and implausible” (2019, p. 48). He grants the importance of the body and of situated activity for cognitive functioning, but he believes that these are explanatorily incomplete without reference to internal information processing. As he puts it, “Humans are indeed embodied dynamic systems embedded in their environments, but our success in those environments depends heavily on our ability to represent them mentally and to perform computations on those representations” (2012, p. 155). Ultimately, for Thagard as for other critics, embodiment is not a viable alternative to the computational-representational paradigm, but merely a dimension of cognitive phenomena that is to be accommodated into information-processing models. Crucially, this leads Thagard to complete skepticism about the use of non-representational embodied accounts for advancing our understanding of creativity in the cognitive science of science: as he affirms categorically, “no one has a clue how to use pure embodiment to explain creative developments in science and technology” (2012, p. 155) because, ultimately, he thinks that “explaining our cognitive capacities requires recognizing representational/computational abilities that outstrip embodied action” (p. 56).

## 3. The Functionalist Roots of Embodied Cognitive Science

The general aim of this article is to propose that, despite its non-representational character, embodied cognitive science (or “4E Cognition”) can make a positive contribution to the goals of the cognitive science of science, including the particular project of making sense of scientific creativity. As suggested at the beginning of the paper and exemplified by Thagard's criticism, this is a controversial proposal. It is controversial because of how the difference between computational-representational cognitive science and embodied cognitive science is often construed. As just seen, computational-representational cognitive science acknowledges the importance of bodily activity at the input and output levels and it investigates the informational states and manipulation processes that lead from sensory input to behavioral output; sometimes it also assigns a role to the body in the specific formatting of representational structures (refer to, e.g., Barsalou, 2008). In contrast, non-representational embodied cognitive science, as seen by critics, appears merely to deny the existence of internal informational states and computational procedures, and to thereby place all the explanatory burden on bodily activities at the input and output levels, in effect leaving a vacuum between the two. This oversimplification is clear, for instance, in the way critics sometimes disparagingly treat the non-representationalism of ecological psychology “as merely a form of dressed-up behaviorism,” as Shapiro (2010, p. 38) puts it in explicit reference to Fodor and Pylyshyn (1981). Bunge and Ardila (2012) also endorse this crude comparison when they state that “Both hold that behavior is the sole effect of environmental stimuli; both model the organism as an empty box. The difference between them is that, whereas ecological psychology focuses on perception, behaviorism is mainly interested in overt behavior” (p. 120). Were this a fair characterization of ecological psychology in particular and of embodied cognitive science more generally, the critics' pessimism would be well justified—but this *is not* a fair characterization. To see why, it is helpful to take a larger view of contemporary embodied cognitive science and to understand it in continuity with the functionalist tradition in psychology developed over a century ago by William James and John Dewey^1^.

For the sake of clarity, it is worth noting how the sense of “functionalism” at play here is different from the one used in other contexts. In a different sense, common, especially in philosophy of mind (refer to, e.g., Polger, 2012; Levin, 2018), functional explanation describes psychological states in terms of what they “do” at a level of description that abstracts away from implementation details. For instance, understood functionally, the heart pumps blood, and this is a function that can be implemented using non-biological materials (e.g., an artificial heart). But more than simply being interested in identifying what the functions of a particular psychological state are, functionalism is the view that equates that state's identity with its function: in this view, “what it is to be a psychological state of a certain sort *just is* and *consists entirely of* having a certain function” (Polger, 2012, p. 337). In mainstream computational-representational cognitive science, explanation at the algorithmic level (Marr, 1982) can be seen as functionalist in this philosophical sense insofar as it explains cognition in terms of mental functions that are medium-independent and, at least in principle, “multiply realizable” or implementable in different media (e.g., in the human brain, in artificial intelligent agents, in distributed computational/cognitive systems, etc.). This is not the “functionalism” this article is concerned with. The psychological approach of James and Dewey is “functionalist” in a different sense, rooted in a Darwinian, naturalist perspective that seeks to understand psychological phenomena in terms of their biological role in nature (refer to, e.g., Godfrey-Smith, 1996; Chemero, 2013). Along these lines, Heidbreder (1933) emphasizes how, for psychological functionalists like James and Dewey, to properly understand a given “mental operation,” it is necessary to “investigate both its antecedents and its consequences, to discover what difference it makes to the organism, and to take into account its whole complex setting in the complex world in which it appears” (p. 202). Psychological functionalism (our focus here) can thus be seen as moving in the opposite direction of philosophical functionalism, rejecting abstract characterizations of mental phenomena and emphasizing their inherently situated, context-dependent, and biological nature. In more contemporary terms, functionalism (in the relevant psychological sense) has turned into a focus on the embedded, enactive, and ecological nature of cognition as a feature of brain-body-environment systems.

Taking into consideration how radically different the functionalist paradigm is makes it possible to move beyond purely negative characterizations of embodied cognitive science (i.e., merely as the rejection of representations) and to arrive at a fuller appreciation of its potential given the resources at its disposal. Toward this goal, the current section offers a selective overview of key aspects of the functionalist framework lying at the foundation of contemporary embodied cognitive science. Besides situating the contemporary work in its proper historical context, this exercise also helps reveal the richness of embodied cognitive science and the resources it has at its disposal for making sense of complex cognitive phenomena including even those at play in scientific creativity. To be clear, however, creativity only becomes our primary focus in section 4: for now, our analysis is at the level of the assumptions at play in psychological theorizing, which includes but is not limited to theorizing about creativity. To have a good grasp of the functionalist paradigm developed by James and Dewey, before examining directly how they construed “mind” and “thought” (section 3.2), it is helpful first to consider how their ideas relate to, and diverge from, popular views stemming from classical empiricism.

### 3.1. Empiricism Without Elementarism and Associationism

In agreement with Locke, Hume, and other empiricists, functionalists in the Jamesian and Deweyan sense see experience as the bedrock of our mental lives and the only possible source of content for our thoughts: the crucial difference is that functionalists reject the assumption of elementarism and associationism. For classical empiricists, thought is built up from “ideas” or “impressions” given by the senses. Our sensory organs register how phenomena in the external world impinge upon them, providing the mind with distinct sensations of simple qualities of objects, such as size, shape, texture, color, tone, taste, smell, and so on. These simple sensory impressions furnish the mind with elements that, in turn, can get associated in various ways. In perception, these elements combine to provide the unitary, coherent experience we have of a tomato, for instance, rather than disconnected impressions of seeing red and of smelling and tasting a peculiar, fresh fragrance and flavor. Beyond perception, however, the association of elementary sensory ideas or impressions is also what explains our ability to think about what we have not seen: if, for instance, I had never seen a yellow or green tomato but you described one to me, I would be able to imagine it by rearranging sensory impressions I had of tomatoes along with non-tomato-related impressions of yellow or green. For classical empiricists, it was important to emphasize that even the most abstract thoughts we may have must originate in experience: in contrast with their rival rationalists (most prominently those of Cartesian influence), who believed that at least some, and perhaps most or all, mental content is innate, empiricists instead held that anything we can think of necessarily traces back to sensory impressions or their combinations, however complex the combination may be.

Like the classical empiricists, James, Dewey, and other functionalists reject nativism and see experience as being of central importance for thought, the only source of what we can think about. But unlike classical empiricists, functionalists reject the assumption of elementarism and associationism, and a straightforward reason for this is that this assumption gets the actual order of events exactly in reverse. As James emphasizes, we perceive objects in their entirety, and the ability to categorize and distinguish the parts and features of objects is added upon the basic experience rather than being its starting point. During childhood, we are taught by adults how to name different characteristics of objects, and through this practice, we develop skills in differentiating between characteristics of a given type as well as among types of characteristics. To use James's own example, in drinking a lemonade on a hot summer day, one can identify “the sourness, the coolness, the sweet, the lemon aroma, etc., and the several degrees of strength of each and all of these things” (James, 1890/1983, p. 652), yet, even if the lemonade is prepared by combining ingredients with these distinctly identifiable characteristics, it is a mistake to conclude that the experience of lemonade flavor is a composite of these elementary sensations. A child drinking lemonade for the first few times experiences a single, simple flavor and only later learns to discriminate the various specific characteristics just described. This results in a qualitative change, “the experience [now] falling into a large number of aspects, each of which is abstracted, classed, named, etc.” (ibid.) and, as such you might judge this lemonade to be too sweet or too sour or too cold. Still, we have no reason to suppose, just because the lemonade was made by combining lemon with sugar and cold water in some ratio, that the experience of the lemonade itself is similarly composed of the addition of independently experienced elementary sensations^2^.

In Dewey, also, we find much the same way of thinking. Already in his classical article against the “reflex arc,” Dewey (1896) emphasizes how the notions of “stimulus” and “response” are analytical categories that the psychologist uses to *describe* phenomena rather than components of the phenomenon *itself* that are separable from each other and independent from observation. Something counts as a stimulus only retroactively, and only because the individual (the observer) had identified some later event to be a response to that earlier event: where no action is observed and labeled as a response, no earlier event is identified as a stimulus, even though there is always a multitude of things seen, heard, touched and so on. So it is not right to say that stimuli cause responses because, quite in the opposite direction, it is our calling something a response that causes us to call something else that preceded it a stimulus. And the same general principle applies to elementarism and associationism, namely, that we have to resist the impulse to reify our analytical categories. This is one version of what James calls the psychologist's fallacy, in which the psychologist as an external observer confuses “his own standpoint with that of the mental fact about which he is making his report” (1890/1983, p. 195). This fallacy manifests itself in a variety of ways, such as in assuming that whatever the psychologist has words to describe must exist, and whatever there are no words to describe must not exist; and assuming that the smallest and/or simplest parts the observer can identify in the phenomenon in fact correspond to its elementary building blocks. As Dewey explains, we must be careful not to assume that the elements we identify when we describe a phenomenon and discriminate its characteristics actually correspond to basic “sensory data” (i.e., simple qualities given to the senses) that combine to yield a unitary perception: “Sensory data, whether they are designated psychic or physical, are thus not starting points; they are the products of analysis” (Dewey, 1925/1929, p. 144). Put differently, the version of the psychologist's fallacy at play here is that of assuming that, because the psychologist can name different parts and aspects of a given object to analytically decompose it, a subject's perception of that same object must be caused by the combining together of such parts and aspects as discrete sensory data. Strictly speaking, in terms of logical or metaphysical necessity, it does not follow from a person's ability to decompose an *object* into parts and features that the person's *thought about the object* is made up from, or decomposable into, (sub)thoughts about its parts and features.

For James and Dewey, then, elementarism and associationism reverse the natural order of events and mistake analytical categories (which are learned) for proper parts of things and of thought. But the more fundamental reason for rejecting elementarism and associationism is that they rest on a problematic assumption about the mind and its relation to the world. This is the assumption that there is a gap between subjects and the “external world” and that experience of the world is indirect, mediated by structures that make present internally (i.e., that re-present) to the mind aspects of what is (or has been) out there. As James neatly puts it, this common but problematic view presupposes that “the mind must in some fashion contain what it knows,” such that, for example, “for *m* and *n* to be known in any way whatever, little rounded and finished off duplicates of each must be contained in the mind as separate entities” (James, 1890/1983, p. 472). Along similar lines, Dewey denounces the common “conception of experience as the equivalent of subjective private consciousness” (Dewey, 1925/1929, p. 11), a conception he sees as motivating a strange conclusion: if appearances stand for and indirectly make present to the mind certain things and events in the so-called “external world,” then what we know and experience directly is only *those appearances* and not real things and events in the real world. In contrast, functionalists understand “experience” as an objective event that unfolds in the real world (which is neither “internal” nor “external”), an event in which, at a particular point in time and space, different processes and objects intersect and relate to one another in various ways. For the human individual, the relations of experience are not only mechanical or chemical, for instance, but also “cognitive,” a term that traditionally was not limited to the “psychological” or “mental” but also encompassed the “epistemic.” In other words, from the perspective of the human individual, experience establishes an objective relation of knowledge, or acquaintance with reality: rather than merely obtaining subjective sensory clues to a mysterious “external” world we are otherwise separated from, having an experience is a matter of participating in a rich web of objective relations through which we directly come to know, or become familiar with, things in the world.

This reconceptualization of experience, or of the mind's relation to the world, imparts a whole new layer of meaning to the functionalists' rejection of elementarism and associationism. When James and Dewey say that experience is not first and foremost of parts and features that are recombined internally but of whole things, their point is not simply that the appearances we supposedly form in “subjective private consciousness” (e.g., sensory inputs, or “ideas” and “impressions”) are holistic, as if representing entire objects before representing their parts. Rather, the point is that, through experience, we become acquainted with entire things (i.e., things themselves, not some supposedly “finished off duplicates” inside the mind), whatever the object of our concern and attention is. No doubt, in many situations, we are especially sensitive to different details and aspects of the objects we are interacting with. Consider, for instance, the difference between buying bananas because you want some fruit (any fruit) for the week to come and, in contrast, buying bananas that are ripe so that you can use them for baking soon, preferably today: in each case, different objects (e.g., bananas compared to apples or cucumbers) and different characteristics of those objects (e.g., their ripeness) will be relevant to you, and will shape your focus and activity. But rather than motivating the conclusion that parts and features are the building blocks of perception, these instances simply illustrate the fact that, in knowing things in their entirety, we can also come to know and care about their elementary parts and features when these matter for specific purposes. As James puts it,

The ‘simple impression' of Hume, the ‘simple idea' of Locke are both abstractions, never realized in experience. Experience, from the very first, presents us with concreted objects, vaguely continuous with the rest of the world which envelops them in space and time, and potentially divisible into inward elements and parts. (James, 1890/1983, p. 461)

This reconceptualization thus makes it possible for functionalists to reject elementarism and associationism while still agreeing with empiricists with regard to the central importance of experience for our mental lives: while classical empiricists assume an agent who is separated from their environment and tasked with piecing together sensory impressions as clues to what may be “out there,” functionalists see experience as an objective relation to entire objects, a relation that can change with attention and intention, but which is of direct, unmediated acquaintance with reality.

### 3.2. Mind and Thought

This radically different functionalist conception of experience, or of our relation to the world, has as its counterpart a corresponding, and equally radically different, conceptualization of mind and thought. In a quote cited above, we saw how James is critical of the view that the mind needs to contain within itself a copy of what it knows—or, put differently, that knowing something amounts to recreating it inside the mind. The key point, now, is that this does not just misconstrue the nature of experience, as just seen, but it also mistakes the nature of mind, assuming it to be the sort of thing that can carry content, that can contain within itself objects of some sort or other.

James illustrates his alternative conception with the image of what he calls the *stream of thought* or *stream of consciousness* (James, 1890/1983). This well-known notion is sometimes interpreted narrowly, as simply emphasizing that thoughts lead to other thoughts in a continuing succession. Although this is not entirely inaccurate, the emphasis on mere succession overlooks the fact that, in favoring the image of the river or stream, James explicitly rejects the railway-inspired image of a “train” constituted by a number of separate, individual cars or wagons lined up one after the other (James, 1890/1983, p. 233). A fuller appreciation of what James meant, then, must take into consideration that, when he first introduced the stream of thought or stream of consciousness, he also offered another name for it that he considered synonymous with the first two: the *stream of subjective life* (p. 233). Subjective life is, to be sure, characterized by a succession: over the course of a person's biography—the historical process that converges and intersects with other historical processes at particular experiences—the individual goes through a succession of activities, which includes a succession of objects interacted with and related to, both physically and conceptually. And as James recognizes, our thought changes over time, between each of these situations, and sometimes even within them. But he was skeptical of the conception of thoughts as discrete entities, each with a different content, and all simultaneously living inside the mind, just waiting to be activated, raised to the level of conscious awareness, or something like that. In line with this, it is worth noting that James usually speaks of a stream of thought (in the singular) rather than a stream of thoughts (in the plural). In the sense at play here, thought is perhaps better understood as the entire mental condition or state of an organism at a given point in time. From this it follows, on the one hand, that *we can never have more than one thought at the same time*: as James puts it, we cannot “have two feelings in our mind at once” (1890/1983, p. 160), there is always a single total state of subjective life at a given point in time. On the other hand, it also follows that *we can never have the same thought more than once*: “no state once gone can recur and be identical with what it was before” (p. 224), to which he adds, “there is no proof that the same bodily sensation is ever got by us twice. What is got twice is the same OBJECT” (p. 225).

These and other points are illustrated in the example mentioned earlier of a child drinking lemonade. James explains:

The mind of the child enjoying the simple lemonade flavor and that of the same child grown up and analyzing it are in two entirely different conditions. Subjectively considered, the two states of mind are two altogether distinct sorts of fact. The later mental state says ‘this is the same flavor (or fluid) which that earlier state perceived as simple,' but that does not make the two states themselves identical. It is nothing but a case of learning more and more about the same topics of discourse or things. (James, 1890/1983, p. 652)

A stream changes over its course: it has its ebbs and flows, and it turns to the right and to the left, sometimes abruptly, other times smoothly. Similarly, the mind (or our thought) changes over time as we attend to, interact with, and come to know better, different objects in different circumstances. Understanding thought in analogy to a flowing stream thus suggests a contrast with the conception of individual thoughts as distinct buckets of water: we *can* get multiple bucketfuls of water out of the stream, conceivably until we emptied out the river bed, yet this doesn't entail that the flowing stream just is a collection of bucket-sized amounts of water bumping into each other; so it is with the mind, whose entire state and whose transition between entire states constitute thought, such that thought cannot be equated to combinations of any kind of discrete, individual sub-parts we may wish to identify. This suggests, further, that *we cannot combine or associate thoughts*, but can only have new thoughts that are informed by previous ones: “Our Thought is not composed of parts, however so composed its objects may be” (James, 1890/1983, p. 344), and “We cannot mix feelings as such, though we may mix the objects we feel, and from their mixture get new feelings” (p. 160). This also relates to the point that the same thought does not occur more than once. You can pick up a bucket of water and walk around with it, then put it back down, and later pick it up again, as many times as you want. The same is possible in some contemporary conceptions of mental content, as illustrated in section 2: once a given content is internally stored, it can be retrieved multiple times, just as it can be used to generate new representations. But if thought is the total state of subjective mental life at a point in time, it makes sense to conclude that we can never go back to a previous state: even if we now interact with and think about the same object as before, this is now a different thought, a different stage in subjective life. The present state is made up of an enriched web of relations and can include, for instance, a different appreciation of the object's meaning, or simply the recognition that this is the same object from earlier (e.g., it is the same lemonade flavor). New thoughts, James says, are “*cognizant* and *appropriative* of the old, whereas the old were always cognizant and appropriative of something else” (1890/1983, p. 335); and, he adds later on, “The knowledge of some other part of the stream, past or future, near or remote, is always mixed in with our knowledge of the present thing” (p. 571). When we think now about the same object as before, the object is the same but the thought is new, partly because it includes in itself prior stages of thought, including our knowledge of the same object back then and including even our knowledge of other objects and events and relations: “Experience is remolding us every moment, and our mental reaction on every given thing is really a resultant of our experience of the whole world up to that date” (p. 228).

Compared to James's idea of the stream of thought—as just seen, so susceptible to misunderstanding—Dewey's conceptualization of mind in linguistic terms is rather straightforward. The dominant conception throughout the history of Western thought has been to treat the mind as a noun, that is, a substance, thing, or object. But thinking is an activity, something that we do; moreover, whatever we do, we can do it more or less mindfully or thoughtfully. Accordingly, Dewey proposes that “mind” is better understood as a verb, an adverb, or an adjective rather than as a noun. Emphasizing the adverbial character of mind, Dewey claims: “*mind* denotes every mode and variety of interest in, and concern for, things: practical, intellectual, and emotional” (Dewey, 1980, p. 263). Later, also highlighting the active, verb-like nature of mind, he states: “Mind is primarily a verb. It denotes all the ways in which we deal consciously and expressly with the situations in which we find ourselves” (ibid.). As such, it is more adequate to see an individual “thought” as a slice of an activity we engage in rather than a distinct entity underlying that activity. Along these lines, Dewey claims: “the organism is not just a structure; it is a characteristic way of interactivity which is not simultaneous, all at once but serial. It is a way impossible without structures for its mechanism, but it differs from structure as walking differs from legs or breathing from lungs” (Dewey, 1925/1929, p. 292). Besides criticizing the reification of the mental and mindful as a supposedly underlying thing (i.e., a mind), this quote also suggests Dewey would agree with James that we cannot have more than one thought at once nor have the same thought more than once. On the one hand, even if we try to multitask, there is always a single thing we are doing, however fast we may be trying to jump between activities and objects of concern. On the other hand, when we repeat an action we have performed before, what that means is typically that we are performing a similar action to accomplish similar ends, just as, by “the same thought” we normally just mean we're once again thinking about the same object. Strictly speaking, however, we cannot have the same thought more than once because we cannot perform the very same action more than once nor move our bodies in exactly the same way as before.

In a passage worth quoting at length, Dewey describes some of the effects of misconceiving mind as a noun, a thing:

Unfortunately, an influential manner of thinking has changed modes of action into an underlying substance that performs the activities in question. It has treated the mind as an independent entity that attends, purposes, cares, notices, and remembers. This change of ways of responding to the environment into an entity from which actions proceed is unfortunate, because it removes the mind from necessary connection with the objects and events, past, present, and future, of the environment with which responsive activities are inherently connected. Mind that bears only an accidental relation to the environment occupies a similar relation to the body. In making the mind purely immaterial (isolated from the organ of doing and undergoing), the body ceases to be living and becomes a dead lump. (Dewey, 1934/1980, pp. 263–264)

Mind and thought are, in the functionalist view, activities we engage in or our manner of dealing with the world—which includes, as seen above, the cognitive or epistemic character of experience for human organisms. It is easy to see how significant this perspective is when it comes to a number of philosophical disputes, including those surrounding the mind-body problem, the problem of other minds, and skeptical concerns about the existence of the external world: in fact, Dewey predicts that “if there were an interdict placed for a generation upon the use of mind, matter, consciousness as nouns” rather than as adjectives and adverbs, we would as a result “find many of our problems much simplified” (Dewey, 1925/1929, pp. 75).

## 4. Discussion: Rethinking Scientific Creativity from a Functionalist, Embodied Perspective

The previous section explored some foundational themes in the functionalist tradition in psychology developed by James and Dewey, emphasizing their views on the nature of mind, thought, experience, and the relation between mind and world. While certainly relevant for an appreciation of the history of the sciences of the mind, this exercise is of more than merely historical interest. The contemporary view of cognition as embodied, embedded, extended, enactive, and ecological may seem to be a recent invention, but its roots extend far back to James's and Dewey's functionalist psychology (on these historical connections refer to, e.g., Heft, 2001; Johnson and Rohrer, 2007; Chemero, 2009; Gallagher, 2009; Lobo et al., 2018; Crippen and Schulkin, 2020; Heras-Escribano, 2021). As such, gaining a better understanding of the functionalist paradigm can be a powerful means to properly understand embodied cognitive science, its goals, and potential. The prospect of developing an embodied cognitive account of scientific creativity seems particularly daunting if—like some critics mentioned in sections 2 and 3—you take embodied cognitive science to hold the same view of mind and thought as mainstream cognitive science *except for the rejection of computation and representation*. But this is a misconception. The functionalist roots of embodied cognitive science reveal a radically different paradigm compared to the one at play in the computational-representational cognitive framework. Considering these differences can provide valuable insight into the field and suggest innovative directions for contemporary discussions about creativity and science. In what follows I sketch this, first, by identifying some direct implications of functionalist ideas for thinking about creativity, and then by linking these to more current views and projects in embodied cognitive science.

Section 2 presented prominent ways of approaching creativity from the computational-representational perspective, in terms of the heuristics a problem-solver creates and uses (Newell, Shaw, and Simon) or, more generally, in terms of how we process and “bind” mental representations of various sorts in the context of everyday activities including but not limited to problem solving (Thagard). Despite the difference in focus and scope, the starting point in both accounts is the view that creative “outputs” arise from manipulations of internal knowledge structures. Of course, this appeal to internal knowledge structures already reveals a point of fundamental disagreement for the classical functionalists and those of us following in that tradition. But it is important not to oversimplify the disagreement. It could perhaps seem like researchers in embodied cognitive science merely and stubbornly refuse to acknowledge the existence of mental representations and internal knowledge structures, as if embodied anti-representationalism was a mere recapitulation of the most extreme forms of behaviorism, as already suggested. More accurately, however, the important point is that the intellectual pull to posit internal knowledge structures arises out of a view of the relation between mind and world that, from the functionalist perspective, is inadequate: namely, the assumption that a gap separates agents from their environment such that we can have no direct acquaintance with reality and can only obtain knowledge *about* the world by reconstructing it internally—as Di Paolo, Buhrmann and Barandiaran put it, this is the problematic assumption that “one cannot have knowledge of what is *outside* oneself except through the ideas one has *inside* oneself” (Di Paolo et al., 2017, p. 23). We saw in section 3 that both James and Dewey reject this view of mind along with the associated view of experience as private subjective consciousness. In line with this, the rejection of elementarism and associationism motivates seeing the binding problem as more of a pseudo-problem. We do not need to explain how representations combine to yield coherent internal “experiences” of the external world: experience (as an objective event that is neither “internal” nor “external”) is itself coherent and connects entire things, such as objects and persons in situations. Knowers can participate in experience more or less fully and more or less attentively, but in any case, the qualitative character of the experience for those participating in it does not arise from combining “ideas” or “impressions” of individual objects or their elementary parts and features, but rather through direct acquaintance with selected aspects of the unfolding situation^3^.

This alternative way of understanding mind and experience has significant implications for how we approach creativity from a psychological standpoint. In an ordinary sense, we say we “have an idea” when we have an insight, when a thought occurs to us that reveals something new, such as a novel plan or course of action. From a Jamesian/Deweyan functionalist perspective, it would be a mistake to think that this amounts to having a new “idea” in the elementarist-associationist sense synonymous with “impressions,” as if a new internal representational structure was created from the combination of previous thoughts (other “ideas” or “impressions”) about the object in question. Thoughts are not countable entities that you can collect and mix and match, but rather are activities we engage in, each constituting a momentary state of subjective life. Similar events may reoccur in life, but one and the same event never literally repeats itself. The same is true of thought. Thinking is something agents do in some situation or other, and that is why, as we have seen, it is impossible to have the exact same thought more than once, just as it is impossible to do exactly the same thing more than once; moreover, we cannot combine or associate thoughts because we do not even have more than one thought at once, no matter how rapidly we try to shift from one thought (i.e., one thinking activity) to another. Every time we think about some object or topic, then, we are having a new thought because we are engaging in a new act of *thinking*, and this is so even if it is the same object or topic as before, and even if we are thinking about it in what seems to be the same way we had previously done. Understood from this functionalist perspective, then, “having an idea” in the ordinary sense amounts to an instance where this thought (which is *new*, as all thoughts always are) involves a significantly changed relation to old objects or topics: through this new thought we now relate to those objects in a different, novel way that is appealing and strikes us as promising.

This motivates reevaluating what we usually call “creativity.” In a way, *every thought is creative*, because every instance of thinking is a new active adaptation to a new situation and experience. On the other hand, we can still speak of activity that is “creative” in the sense of being *distinctly* or *differently* innovative—just what the distinction or difference is, however, must be considered more carefully.

In popular media and in academic circles alike, discussions about creativity, and especially about creativity in science, tend to revolve around examples of extraordinary, even revolutionary innovators like Galileo, Newton, Darwin, Einstein, and a few others. Although understandable and justified in some contexts, this focus on seemingly unique individuals can be misleading. Figures like the ones just mentioned have over time gained a special cultural status because of their influential and innovative ideas. But this personal status sometimes seems to motivate the conclusion that their ideas were special because they were theirs, that the ideas were creative because they were had by creative people, rather than the other way around. The mythical standing of creative geniuses is entangled with the psychologization of “creativity” as an individual mental attribute, whether as a special mental faculty that acts as the source of creative ideas or as a cognitive process that yields creative outputs: either way, as something that people own and possess, creativity also becomes something that people possess *differentially*, that some have more of, others less, and others conceivably lack entirely.

As already seen, researchers studying creativity often point out that what makes some idea or product creative is the fact that it is novel, surprising, and useful, or somehow valuable (e.g., Boden, 2004; Simonton, 2012). This reasonable perspective should raise a red flag for internalist views that hope to find “a single cognitive or neural mechanism” responsible for “the extraordinary creative capacities of an Einstein or a Shakespeare” as Dietrich and Haider (2017, p. 1) put it. These popular criteria suggest that there is nothing intrinsic to a given idea or product that makes it inherently creative: rather, an idea's or product's “creativeness” is a relational characteristic, one that depends in part on how the idea or product in question relates to other ideas or products (e.g., which other ideas or products exist and what they are like) and in part, it depends on how the idea or product in question relates to other people (for whom the new idea or product is more or less useful in some way, for some purpose). From this, it follows that we cannot reasonably expect there to be internal mechanisms that fully explain the origin of creative ideas or products. If the sociomaterial context (at least partly, if not entirely) determines which ideas or products count as creative in that context and which do not, then that means that one and the same idea or product could, in different circumstances, count as creative *and* as not creative. Accordingly, even if there were “cognitive or neural mechanisms” that properly and uniquely explain the origin of certain ideas or products we consider creative *in a given context*, the same mechanisms might not be explanatory of creativity *in other contexts* because the ideas or products they supposedly originate would *themselves* not count as creative in those other contexts. Given the sociomaterially-extended nature of what makes ideas and products count as creative, we reify “creativity” when we see it (only) as an individual mental endowment, a context-independent ability or capacity, and something that the geniuses—the Einsteins, Newtons, and Darwins—possess to an unusual degree, more than the rest of us.

If, in a sense, all thoughts and behaviors are creative, and if what makes *some* thoughts and behaviors *distinctly* creative is, at least in part, the sociomaterial context, then the challenge we are faced with is that of making sense of the specifically psychological or cognitive conditions for these distinctly creative thoughts and behaviors, and to do so without falling prey to tendencies toward internalist reification and individualistic exceptionalism. My proposal is that a promising direction for tackling this question from the perspective of embodied cognitive science lies precisely in expanding upon the radical functionalist conceptions of mind, thought, and experience.

In discussing the functionalist understanding of experience, or of mind's relation to the world, I suggested that attention and intention play a role in the way an individual engages in experience. The point is that an individual may be more or less attuned to experience, more or less sensitive to the relations and unfolding events, and may participate more or less fully in the situation. To elaborate on this point, let us consider a concrete situation such as a lab meeting in which colleagues meet to discuss updates in their scientific research. An extreme case of a shift in your participation in that shared experience would be if you fell asleep for a brief moment: even as your nodding off is part of the objectively unfolding situation and others might even notice it, this is an instance in which the experience temporarily has no subjective qualitative character for you (it lacks a “how” for you, as Dewey puts it). But even while fully awake our attention can shift in different directions, which is another way to frame our participation in experiences. If you get distracted, you might briefly tune out of the discussion and notice the threatening clouds out the window, and from them turn to the parking lot, and then to the plans you had of stopping by the grocery store on the drive home after the meeting, until you realize you are thinking about recipes rather than contributing to the discussion. Alternatively, you may be fully invested in the conversation and your shifts in attention are constructive and contribute to the goal of the discussion: so instead of noticing the clouds and the parking lot out the window, you notice something in the graph projected on the screen, or in your colleague's description of their new experimental design, and by mentioning these you help open up new directions for the discussion. Of course, it could even be a combination of these two scenarios, such as, for instance, if your momentarily turning away to the clouds and the parking lot out the window actually leads you to notice something different that is relevant for the ongoing discussion: maybe a technical difficulty can be avoided by uploading the experimental data “to the cloud” or perhaps the bright green car in the parking lot suggests a different experimental manipulation to try or the car's sudden breaking inspires you to think of a potentially useful tweak in the damping parameter in the mathematical model under discussion. This example intimates two important aspects of the distinctly “creative.”

The first has to do with the relation between insight and sight, or between creativity and perception more generally. In different ways of participating in a shared experience such as the ones just described, “having an idea” that is valuable for others can be the result of an individual attending to different aspects of the situation and, sometimes quite literally, *seeing* different things and/or seeing the same things differently. James seems to suggest as much when he says, “some people are far more sensitive to resemblances, and far more ready to point out wherein they consist, than others are. They are the wits, the poets, the inventors, the scientific men, the practical geniuses” (James, 1890/1983, p. 500). More recently, building upon functionalist foundations, ecological psychologists speak of perception as “an achievement of the individual, not an appearance in the theater of his consciousness” and as “a keeping-in-touch with the world, an experiencing of things rather than a having of experiences” (Gibson, 1979, p. 239). In this ecological perspective, perceiving is most prominently an act of detecting *affordances*, that is, detecting possibilities for action that the environment offers the agent given the agent's skills and sensorimotor makeup in relation to the characteristics of the object or surface in question (Gibson, 1979; Reed, 1996; Heft, 2001; Chemero, 2009; Turvey, 2018). Sometimes individuals just casually happen upon some aspect of the situation that others had not noticed, perceiving affordance others had so far overlooked. But individuals can also over time become differentially sensitive to some aspects of the situation more than others (that is, more than they are sensitive to other aspects and also more sensitive than other people are to the same aspects): as an activity, perceiving can become more skillful through attunement or the “education of attention” (Gibson, 1966; Jacobs and Michaels, 2007; Araújo and Davids, 2011). This can be seen as a specific instance of what James and Dewey both referred to as “habits,” that is, specialized, context-sensitive patterns of activity—in this case, the activity of perceiving or exploring the environment (refer to James, 1890/1983, 1899; Dewey, 1922; Segundo-Ortin and Heras-Escribano, 2021; for related embodied accounts emphasizing the ecology of skillful performance refer to, e.g., Christensen et al., 2016; Christensen and Sutton, 2019; Sutton and Bicknell, 2020). Importantly, however, what is at play here is direct contact with reality and the detection (rather than inference or estimation) of relations between things in the world: “The mind does not need to associate sequential regularities; such regularities only need to be detected. Associations are perceived rather than perceptions associated” (Lombardo, 1987, p. 291). Having an insight, then, can be a matter of noticing that some object (physical or conceptual) affords a given manipulation that turns out to be useful in a novel and surprising way.

To emphasize the education of attention, as above, is to emphasize the dynamically changing nature of the perception, or how our engagement with the world and participation in experience can become more skillful over time. This connects to the second point, which has to do with the dynamically changing nature of the creative activity itself. In a recent article focusing on jewelry making, Baber et al. (2019) criticize the notion that a designer begins with a complete idea and then works *on* some material to impose that form onto it; rather, they propose, the designer works *with* materials to discover what the final product can and will be. The authors criticize the common “separation of ‘creation-in-the-head' [from] a physical behavior that is merely the acting out of this ‘cognition'” (p. 285), a view that they see as inadequate because, as they observe, “technology mediates human activity through a process of mutual co-construction” (p. 284). At the beginning of the process, the material presents the jeweler with a range of possibilities but it also imposes specific limitations depending on its particular features, including its unique shape, composition, deformities, etc. As the jeweler engages with that material, each new intervention reveals and creates new possibilities and limitations. Drawing from dynamical systems theory the authors propose, “‘creativity' arises from the dynamic interplay between jewelers and their technology” (p. 284).

What is worth highlighting here is that creative activity (including creative thinking) is not simply a matter of perceiving unusual affordances in the here and now, nor of merely having over time developed skillful perceptual attunement such that we can perceive affordances others cannot: rather, or in addition, the activity itself has this dynamic, co-constructive character, in which each new engagement further constrains and enables future activity, such that the outcome cannot be fully articulated in advance, before this iterative, interactive process started. In the case of creativity in science, this includes, of course, literal physical manipulations through which we operate in an innovative way on an object (e.g., a measuring instrument, or the thing measured) or combine objects in an unusual way. But it can also include the manipulation of symbolic structures, such as variables in a mathematical formula or concepts in a linguistically-articulated theoretical description, in writing, in conversation, or in soliloquy. In this perspective, what is distinct about the psychological dimension of creative breakthroughs in science is the especially attuned or “educated” attention some individuals develop which allows them not only to see what others cannot or do not, but also to engage with materials, objects, and structures (including symbolic ones) in ways that dynamically co-create further possibilities for action (i.e., affordances).

These references to the conceptual and symbolic might strike some readers as a case of smuggling in representational thinking through the back door. On the contrary, however, I see the present paper as contributing to the larger project many of us in embodied cognitive science are already engaged in of not simply responding to the ‘scaling up challenge' but in fact questioning the representation-hunger often ascribed to some cognitive phenomena (refer to, e.g., Zahnoun, 2021). Along these lines, it is useful to point out how the perspective on creativity being sketched here resonates with recent work approaching concepts and conceptual thought in ways that challenge more traditional internalist, representational accounts. also, e.g., Aerts et al., 2013). From a very different theoretical starting point, with a more explicitly cognitive neuroscientific focus, but moving in a similar direction, Casasanto and Lupyan (2015) provocatively propose that “all concepts are *ad hoc*,” by which they mean that concepts are situation-specific, on-the-fly behavioral adaptations that “differ from one instantiation to the next, within and between individuals and groups” and that “are shaped by the contexts in which they are instantiated on every time scale, from the millisecond to the lifetime” (p. 561). In their view, we should move away from theorizing about “concepts, categories, and word meanings as things that we *have in* our minds” and instead “build theories of conceptualizing, categorizing, and constructing word meanings: things that we *do with* our minds” (Casasanto and Lupyan, 2015, p. 543, emphasis original).

Other recent contributions emphasize the inherently social nature of conceptual thought as grounded in language, itself understood as an embodied social practice in the first place. Along these lines, Contreras Kallens et al. (2018) describe categorization as a social, interpersonal phenomenon that is flexible and can change over time, being even subject to cultural evolution and, therefore, amenable to modeling at the population level using similar methods already applied to other phenomena in cultural evolution. Through more anthropological and archaeological lens, John Sutton and colleagues have offered a compelling picture that emphasizes the inextricably material and social dimensions of skillful, creative, and conceptual activities, including the distributed cognitive activities at play in remembering (e.g., Sutton, 2008, 2015, 2020; also Sutton et al., 2010; Sutton and Keene, 2016). And from an individual developmental standpoint, Heft (2020) highlights how the sociocultural specificity of developmental trajectories makes it so that individual perception-action is never socioculturally ‘neutral', but is by necessity fundamentally shaped by shared practices: naturally, this includes linguistic practices, such that naming and talking about complex abstract processes or systems (e.g., the postal system) not only enables conceptual thinking, but makes it possible for conceptual thinking to guide adaptive perception-action in the here and now.

What these admittedly diverse lines of research reveal is that reference to the conceptual and symbolic, as in the present paper, need not amount to capitulation to internalist, representationalist theorizing. The view of creativity being sketched here focuses specifically on perception-action to propose that we understand creative innovation in terms of the enhanced perception of affordances (i.e., the educated attention) and the co-construction of possibilities through active engagement, that is, in terms of what Heft (2020) calls “habits of attention and skilled action” (p. 823). Still, this focus on perception-action is perfectly compatible with the recognition that behavior, as shaped by development within specific sociocultural practices, involves abilities to perceive-act that are sensitive to conceptual and symbolic meaning. In fact, this is something that becomes even clearer when we take into account the functionalist foundations of contemporary theorizing in embodied cognitive science, and in particular, the way functionalists made sense of intelligent behavior and the emergence of novelty, including the especially creative, in complex human practices such as those we find in science.

As Dewey puts it, “Command of scientific methods and systematized subject-matter liberates Individuals; it enables them to see new problems, devise new procedures, and, in general, makes for diversification rather than for set uniformity” (Dewey, 1929, p. 12). Just as our current thought is shaped by prior thoughts, experience informs further engagement with the world by guiding what and how we see; this, in turn, as Dewey emphasizes here, further shapes understanding, or our ability to engage meaningfully with the world. So, Dewey adds, “Because the range of understanding is deepened and widened [the scientist] can take into account remote consequences which were originally hidden from view and hence were ignored in his action”; as a consequence, “Seeing more relations he sees more possibilities, more opportunities. He is emancipated from the need of following tradition and special precedents. His ability to judge being enriched, he has a wider range of alternatives to select from in dealing with individual situations” (Dewey, 1929, p. 20-21). The specificity of experience shapes personal development, making some individuals sensitive to different aspects of the situation, and enabling them to detect and articulate unusual relations to and between objects, facts, and events; this also enables individuals to, by working with objects, give rise to new possibilities that they can then perceive and act on. In this way, creative developments in science can arise from an individual's differential sensitivity to existing possibilities and, through active engagement in the world, the co-creation of novel possibilities—all of which embody (or in-*habit*) sociocultural practices, including practices of naming things and using language to constrain (limit and enable) perception-action in particular ways.

Building upon the functionalist foundations of embodied cognitive science, then, motivates approaching creativity as an activity rather than an underlying capacity, and understanding it psychologically but non-representationally in terms of affordance perception, the education of attention, and co-constructive embodied engagement in experience. No doubt, an approach like this constitutes a departure from traditional cognitive views that favor explanation in terms of internal, individual computational mental mechanisms. But, in this picture, creativity is still “cognitive” in the deeper sense that goes beyond the “psychological” and includes the “epistemic”: creativity is a matter of *knowing* reality, being acquainted with aspects of the world, and (re)cognizing possibilities, including even dynamically changing ones. In this sense, creativity is clearly dependent on previous knowledge, yet this does not entail anything representational: to know something is not to construct an internal copy of it, but rather to undergo transformation through experience, to have our modes of activity (including thinking) changed by transaction with things in the world. The role of the imagination in some creative activities (e.g., involving abstract reasoning) need not be seen as problematic. As Dewey (1934/1980) suggests, the ideas we come up with through imagination “are not made out of imaginary stuff. They are made out of the hard stuff of the world of physical and social experience” (p. 49). He adds: “The locomotive did not exist before Stevenson, nor the telegraph before the time of Morse. But the conditions for their existence were there in physical material and energies and in human capacity. Imagination seized hold upon the idea of a rearrangement of existing things that would evolve new objects” (p. 49). In this embodied framing, then, creative activity is cognitive in that it is a matter of expanding our knowledge and appreciation of what is possible and what could be made possible through interventions and interaction. The special and distinctly creative insight, then, is not a mental impression triggered in subjective consciousness but a specialization of thinking activity that is enriched because of what it knows. And this, again, is the the result of learning, that is, changing with experience, or the formation of habits:

The reason a baby can know little and an experienced adult know much when confronting the same things is not because the latter has a “mind” which the former has not, but because one has already formed habits which the other has still to acquire. The scientific man and the philosopher like the carpenter, the physician, and politician know with their habits not with their “consciousness.” The latter is eventual, not a source. Its occurrence marks a peculiarly delicate connection between highly organized habits and unorganized impulses. (Dewey, 1922, p. 182–183)

Through experience, over time, we develop specialized ways of doing things and relating to the world (including both material and conceptual objects) that are sensitive to the context and task we are engaged in. Learning scientific methods and facts, then, like other specializations, is a matter of developing specific ways of dealing adaptively with particular situations. In this context, creative insight is a cognitive accomplishment in that it concerns epistemic contact with reality and the discovery of what the world presents to us.

To conclude, we are now in a different position to reconsider the view of creativity as the ability to combine or associate old, familiar ideas to generate new ones. If interpreted in traditional computational-representational terms, the combinatorial view commits a version of the psychologist's fallacy. No doubt, creative products—a novel mathematical model or a novel theoretical formulation that a scientist may come up with—are created by combining other previously existing physical and conceptual materials. But it is a fallacy to conclude from this that the *idea* of the product must have originated through the combination of the *ideas* of those other products and their parts or aspects. In creative activity, we do combine old, familiar things in new ways, but that is not detached from embodied engagement with the world around us: “The new vision does not arise out of nothing, but emerges through seeing, in terms of possibilities, that is, of imagination, old things in new relations serving a new end which the new end aids in creating” (Dewey, 1934/1980, p. 49). In science as in other specialized domains, creative breakthroughs emerge from skilled, habituated sensorimotor activity in which we detect and forge, with old and familiar physical or conceptual objects, possibilities for further adaptation in a dynamically changing experience. One example is precisely what we are doing right now: revisiting old theories and methods—here, ideas about mind, thought, and experience from the functionalist tradition—as a way to inform and re-orient how we act now, including how we think about and address the scientific challenges we are faced with. If science involves standing on the shoulders of giants, then creative activity can be seen as a matter of sensitivity to the situation and perceiving whose shoulder to climb and which direction to look so as to go from the old to the new, casting fresh light on current problems and, through that, setting up new paths for advancing in understanding.

## 5. Conclusion

In the context of current debates about the limits of embodied cognitive science, this article offered an entry point to considering the embodied approach's promise for contributing to research in the cognitive science of scientific creativity. A common view of creativity describes it in combinatorial terms as involving associating what is old and familiar in order to generate something novel. One example of a combinatorial account of creativity in the cognitive science of science was examined, highlighting the role it assigns to the processing of internal knowledge structures for generating creative outputs. Proponents of the computational-representational paradigm criticize embodied cognitive science for being unreasonable and implausible in its anti-representationalism—hence the challenge to ‘scale up' embodied explanations to account for ‘representation-hungry' phenomena. Accordingly, the article was concerned with providing a richer picture of embodied cognitive science, moving beyond merely negative characterizations (i.e., simply as a rejection of representations). The article did this by exploring contemporary embodied cognitive science's theoretical and conceptual roots in the functionalist tradition in psychology developed by William James and John Dewey. As a continuation of this functionalist tradition, contemporary embodied cognitive science inherits a rich and sophisticated way of thinking about mind, thought, and experience, or the relation between mind and the world, one that is radically different from the computational-representational paradigm, but that amounts to more than a mere rejection of representations. For these classical functionalists, “mind” is something we do, or rather the character of some of our activities (e.g., when we act thoughtfully or mindfully). Not only that, but “thought” is similarly reframed as a total (if momentary) state of subjective life, or a slice of a dynamically unfolding activity (namely, thinking). A consequence of this view is that we cannot have more than one thought at once, nor have the same thought more than once, nor combine thoughts—which casts doubt on the combinatorial view of creativity interpreted in internalist, computational-representational terms. After identifying these key foundational aspects of the functionalist inheritance of embodied cognitive science, the article offered a sketch of how these concepts translate into a contemporary understanding of creative innovation in science. Focusing primarily on perception-action, the sketch offered here described creative innovation in terms of enhanced affordance perception (through the education of attention) and the dynamical co-construction of possibilities through active engagement with material and conceptual resources. The relation between this sketch and recent work on concepts and conceptual thinking, on the one hand, and between the two and old functionalist ideas, on the other, was explored in order to reveal how even a perception-action-focused view of creative innovation such as this can accommodate engagement with conceptual and symbolic resources in creative thinking and problem solving. The result is a view that, while not computational or representational, is still “cognitive” in that it sees creativity as a matter of *knowing* reality, being acquainted with aspects of the world, and (re)cognizing possibilities. Although it is in tension with internalist interpretations of the combinatorial view, the sketch offered here in itself illustrates how we combine old ideas (in this case, from the classical functionalists) in different contexts to respond to novel challenges and find creative solutions to new problems. Creativity involves combination and goes from old to new, but it is just not something that is done in our heads: rather, creativity is a feature of the mind's *relation* to the world, which is what the functionalists called “experience” and what we today describe in terms of brain-body-environment systems.

## Data Availability Statement

The original contributions presented in the study are included in the article/supplementary material, further inquiries can be directed to the corresponding author.

## Author Contributions

The author confirms being the sole contributor of this work and has approved it for publication.

## Funding

I acknowledge support by the German Research Foundation and the Open Access Publication Fund of TU Berlin.

## Conflict of Interest

The author declares that the research was conducted in the absence of any commercial or financial relationships that could be construed as a potential conflict of interest.

## Publisher's Note

All claims expressed in this article are solely those of the authors and do not necessarily represent those of their affiliated organizations, or those of the publisher, the editors and the reviewers. Any product that may be evaluated in this article, or claim that may be made by its manufacturer, is not guaranteed or endorsed by the publisher.

## Footnotes

^1^On the specific question of the relation between ecological psychology and behaviorism, including their similarities and differences, more nuanced discussions can be found in, e.g., Reed (1996), Heft (2001), and Barrett (2016).

^2^This is an issue I will address directly later (section 4), but for now the reader is invited to consider how this example from James and the point articulated here relate to the binding problem discussed in section 2.

^3^It's worth noting that, despite the radical theoretical and conceptual differences between the paradigms, the functionalist embodied framework is not inimical to the applied, practical orientation that traditional cognitive science has had since its early days, of pursuing explanations of cognitive phenomena by building mechanisms that implement certain principles (as seen in Newell, Shaw and Simon's work). In this direction, one reviewer asks how we could build something that has ‘direct acquaintance with selected aspects of the unfolding situation'. The simplest and most concrete answer is to point in the direction of applied work that already does that, such as work in evolutionary or biologically-inspired robotics (e.g., Brooks, 1990, 1991; Webb, 1995; Reeve et al., 2005) and in artificial life (e.g., Beer, 1997, 2004, 2020; Froese et al., 2014; Campos and Froese, 2019). In more traditional approaches, it is common to model cognitive agency in terms of an input-output system with internal procedures that are responsible for actively controlling movement or activity (e.g., selecting, initiating, evaluating and correcting the execution of motor programs) and for interpreting incoming signals to build and update internal models of the environment and the likely sources of stimulation. In contrast, a common theme in projects in evolutionary robotics and artificial life such as the ones just cited is the emphasis on modeling cognitive agency in terms of intrinsic activity and selective sensitivity to the environment, where activity and sensitivity constrain and guide each other, both limiting and enabling a range of specific ways of direct participation in, and adaptation to, the unfolding situation.
